# Unequal Trends in Coronary Heart Disease Mortality by Socioeconomic Circumstances, England 1982–2006: An Analytical Study

**DOI:** 10.1371/journal.pone.0059608

**Published:** 2013-03-20

**Authors:** Madhavi Bajekal, Shaun Scholes, Martin O’Flaherty, Rosalind Raine, Paul Norman, Simon Capewell

**Affiliations:** 1 Department of Applied Health Research, University College London, London, United Kingdom; 2 Institute of Psychology, Health and Society, University of Liverpool, Liverpool, United Kingdom; 3 School of Geography, University of Leeds, Leeds, United Kingdom; University of Oxford, United Kingdom

## Abstract

**Background:**

Coronary heart disease (CHD) remains a major public health burden, causing 80,000 deaths annually in England and Wales, with major inequalities. However, there are no recent analyses of age-specific socioeconomic trends in mortality. We analysed annual trends in inequalities in age-specific CHD mortality rates in small areas in England, grouped into deprivation quintiles.

**Methods:**

We calculated CHD mortality rates for 10-year age groups (from 35 to ≥85 years) using three year moving averages between 1982 and 2006. We used Joinpoint regression to identify significant turning points in age- sex- and deprivation-specific time trends. We also analysed trends in absolute and relative inequalities in age-standardised rates between the least and most deprived areas.

**Results:**

Between 1982 and 2006, CHD mortality fell by 62.2% in men and 59.7% in women. Falls were largest for the most deprived areas with the highest initial level of CHD mortality. However, a social gradient in the pace of fall was apparent, being steepest in the least deprived quintile. Thus, while absolute inequalities narrowed over the period, relative inequalities increased. From 2000, declines in mortality rates slowed or levelled off in the youngest groups, notably in women aged 45–54 in the least deprived groups. In contrast, from age 55 years and older, rates of fall in CHD mortality accelerated in the 2000s, likewise falling fastest in the least deprived quintile.

**Conclusions:**

Age-standardised CHD mortality rates have declined substantially in England, with the steepest falls in the most affluent quintiles. However, this concealed contrasting patterns in underlying age-specific rates. From 2000, mortality rates levelled off in the youngest groups but accelerated in middle aged and older groups. Mortality analyses by small areas could provide potentially valuable insights into possible drivers of inequalities, and thus inform future strategies to reduce CHD mortality across all social groups.

## Introduction

Coronary heart disease (CHD) remains a major public health burden in England and Wales, causing 80,000 deaths in 2007 and chronically affecting about 3 million patients. Major inequalities have been described, with marked social and regional differences in premature mortality rates [1]–[4].

The rate of mortality decline has usually been described in terms of overall age adjusted rates, and little attention has been paid to differentials in terms of age and socioeconomic characteristics. Yet, several recent reports suggest that despite striking age adjusted declines, a slowing or plateauing in mortality rates has been observed in young adults in the last decade of the twentieth century. The flattening of CHD mortality rates in young adults has now been reported in England & Wales [5], the United States, Australia [6]–[7] and New Zealand [8]. A marked reduction in healthcare use is implausible. The spotlight falls on recent changes in major cardiovascular risk factors, especially dramatic increases in obesity and diabetes in most industrialised countries [9]–[10], flattening of previous blood pressure falls in US women [11]–[12] and persistent smoking in young adults in the United Kingdom and elsewhere [13]. The slowing of the decline in mortality in young adults was also observed in Scotland, but limited to young men living in the most deprived areas [14].

It remains uncertain whether the observed pattern of a slowing down in the pace of fall in CHD mortality amongst young people continued into the 21^st^ century. Nor whether, as in Scotland, the flattening in mortality trends amongst young people has been confined to socially deprived groups. There are no reports of age-specific socioeconomic trend differentials in England. Our aim was therefore to analyse recent patterns in age, sex and socioeconomic circumstances specific CHD mortality trends in England during the period 1982–2006.

## Methods

### Data

We used the Index of Multiple Deprivation 2007 (IMD) as our indicator of socioeconomic circumstances [15]. The IMD is a measure of multiple deprivation at the small area level, the Lower-layer Super Output Area (LSOA), an area covering an average of 1,500 people. The overall IMD score is a weighted composite of indicators measuring seven domains of deprivation (income; employment; health and disability; education, skills and training; barriers to housing and services; crime; living environment). The 32,482 LSOAs in England were grouped in equal fifths according to ranked IMD score, quintiles one and five (Q1 and Q5) representing the least and most deprived areas respectively. The IMD quintile group membership of an area remained fixed over the period of analysis, ie 1982–2006. In other words, we compared trends for the same set of areas across all years of the study. When classified into deprivation quintiles, the relative ranking of areas remained markedly stable over this period (see supporting information Text S1). At this level of aggregation (6.5 million adults aged 35 years or older and 6.5 thousand LSOAs per quintile), selective net migration between quintile groups was reckoned to have some, but not a significant impact on the analysis of trends reported [16].

Corresponding LSOA mid-year population estimates by five year age-group and sex for the period 2001–07 were provided by the Office for National Statistics (ONS) as ‘experimental statistics’. For 1981–2000, we estimated populations by extending previous work using a cohort-component model with outputs constrained to sum to the ONS subnational estimates for each year [17]. All age-sex population estimates were aggregated into the deprivation quintiles described above.

We obtained mortality data by year of registration of death for the period 1981 to 2007 from ONS. For each year, ONS provided counts of deaths aggregated up to 3-digit International Classification of Diseases (ICD) codes in five year age bands by sex for each of our five deprivation groups. We determined underlying cause of death from coronary heart disease by selecting on ICD-9 (ninth revision) codes 410–414 for the period 1981–2000 and ICD-10 (tenth revision) codes I20–I25 for 2001–07.

When segmented by sex, 10-year age bands and deprivation quintiles, age-specific CHD death rates were subject to random annual fluctuations, particularly in the younger ages. To reduce variability and capture the underlying trend, we calculated age-specific rates using three year moving averages. We quote just the central year to denote the rate for each three-year interval (ie ‘1982’ for rates calculated by pooling mortality and population data over the years 1981, 1982 and 1983). Hence the series analysed, or our study period, runs from 1982–2006: but we also used data for the years 1981 and 2007 to derive the first (‘1982’) and last (‘2006’) data points in the series.

We restricted our analyses to persons aged 35 years and older, with those aged 85+ comprising the last open-ended age band. Overall CHD mortality rates by deprivation quintile were age-standardised using the European standard as the reference population. Age-specific mortality rates and their standard errors by sex, year, and deprivation quintile are available as supplementary information (Table S1 (men), Table S2 (women)).

### Statistical Analysis

We used the Joinpoint Regression Programme (version 3.4.2, Oct 2009) to estimate periods with similar annual percentage change in mortality rates. We used a Bayesian information criterion approach and allowed a maximum of three join points (i.e. four segments) with a minimum of four data points per segment so as to include at least one non-overlapping data point in a segment composed of three-year moving averages. In addition to the annual percentage change (APC) over each segment, Joinpoint also calculates a weighted average annual percentage change (AAPC) over the whole 25 years of the study.

We measured inequality along two axes: the absolute difference or the gap between the mortality levels in the most and least deprived quintiles; and a relative measure of inequality expressed as a ratio between the top and bottom quintiles. We have presented these two axes based on simple calculations as well as on regression analyses. The simple measures are the age-standardised rate difference and rate ratios of the direct estimates for the two extreme fifths of the population. The regression-based analysis takes into account the values across the whole spectrum of deprivation quintiles and the population size within each quintile in every year. Corresponding to the simple measures, the slope index of inequality (SII) provides the equivalent absolute difference in age-standardised mortality rates and the relative index of inequality (RII) provides a measure of ratio of the estimated health of the most deprived person compared to the estimated health of the least deprived person in the population. Thus, even though the deprivation quintiles were based on roughly equal population sizes, the simple inequality estimates which compared values for the extreme *fifths* of the population were less wide than the regression-based estimates which estimated the inequality between the hypothetical *persons* at each end of the distribution. We calculated the SII and the RII using the Health Disparities Calculator (HD*Calc version 1.2.2, June 2012). For the RII, we have used the Kunst-Mackenbach Index (KMI) as this is conceptually closest to the simple rate ratio measure [18].

## Results

### Overall Change in Age-adjusted CHD Mortality Rates

Between 1982 and 2006, the age-standardised rate for CHD mortality in England fell by about 60% in men and women (62.2% and 59.7%, respectively). Rates declined slightly faster for men, averaging 4.0% per year, than for women (3.7% per year) (Table 1). However, absolute rates remained over twice as high for men compared to women throughout the period. Thus CHD mortality rates in men reached a similar level in 2006 (272 per 100,000, *95% confidence limits: 270.7, 273.7* per 100,000) as rates in women more than a decade previously (280 (*278.3, 281.0*) per 100,000 in 1992).

**Table 1:** Age-standardised CHD mortality rates per 100,000 by deprivation quintiles and sex, England 1982–2006.

	Year		Least Deprived	Q2	Q3	Q4	Most Deprived	England	Gap (Q5-Q1)	Rate Ratio (Q5/Q1)	95% CI for Rate Ratio	Slope Index of Inequality	Relative Index of Inequality^1^	95% CI for RII
**MEN**	**1982**		577.8	645.8	702.5	778.3	878.3	719.5	300.5	1.52	*(1.50,1.54)*	366.1	1.69	*(1.683,1.688)*
	**1983**		568.0	639.9	697.0	773.0	878.7	713.8	310.7	1.55	*(1.53,1.57)*	376.3	1.72	*(1.716,1.722)*
	**1984**		562.6	639.5	699.2	778.3	886.2	715.2	323.6	1.58	*(1.56,1.60)*	391.6	1.76	*(1.755,1.760)*
	**1985**		552.0	627.4	686.6	768.7	880.8	704.6	328.8	1.60	*(1.58,1.62)*	397.5	1.79	*(1.787,1.793)*
	**1986**		537.2	611.6	669.7	750.5	867.4	688.1	330.2	1.61	*(1.59,1.64)*	397.1	1.82	*(1.813,1.819)*
	**1987**		518.3	586.4	639.8	718.0	839.3	660.4	321.0	1.62	*(1.60,1.64)*	383.4	1.82	*(1.820,1.826)*
	**1988**		494.2	563.6	620.7	690.2	815.9	636.2	321.7	1.65	*(1.63,1.67)*	381.0	1.86	*(1.857,1.863)*
	**1989**		476.0	546.3	596.6	667.6	795.5	615.1	319.5	1.67	*(1.65,1.69)*	375.3	1.88	*(1.881,1.887)*
	**1990**		462.5	533.2	586.7	654.9	777.0	601.0	314.5	1.68	*(1.66,1.70)*	370.3	1.90	*(1.893,1.900)*
	**1991**		453.3	519.9	568.3	641.9	759.7	586.1	306.4	1.68	*(1.65,1.70)*	361.6	1.90	*(1.895,1.901)*
	**1992**		448.8	510.0	560.0	631.7	746.9	576.3	298.1	1.66	*(1.64,1.69)*	352.8	1.89	*(1.884,1.890)*
	**1993**		435.4	487.7	533.8	603.7	720.6	552.5	285.3	1.66	*(1.63,1.68)*	336.1	1.88	*(1.876,1.882)*
	**1994**		419.9	468.9	513.6	583.8	693.6	531.8	273.7	1.65	*(1.63,1.68)*	324.0	1.88	*(1.877,1.883)*
	**1995**		398.1	444.0	488.8	555.9	658.8	504.6	260.7	1.65	*(1.63,1.68)*	309.6	1.89	*(1.885,1.892)*
	**1996**		376.7	424.0	468.2	534.5	635.5	482.8	258.8	1.69	*(1.66,1.71)*	307.0	1.94	*(1.932,1.939)*
	**1997**		356.7	405.2	447.7	508.1	612.0	460.5	255.4	1.72	*(1.69,1.74)*	299.5	1.97	*(1.962,1.970)*
	**1998**		336.6	380.9	424.4	482.8	586.1	436.3	249.6	1.74	*(1.72,1.77)*	292.8	2.01	*(2.008,2.015)*
	**1999**		318.9	361.6	403.6	461.0	557.8	414.5	238.9	1.75	*(1.72,1.78)*	281.0	2.03	*(2.022,2.030)*
	**2000**		299.4	340.5	382.8	441.0	531.3	392.6	231.8	1.77	*(1.75,1.80)*	274.6	2.08	*(2.071,2.080)*
	**2001**		281.6	324.8	361.9	424.7	509.6	373.6	228.0	1.81	*(1.78,1.84)*	270.4	2.13	*(2.128,2.136)*
	**2002**		266.1	311.1	343.6	404.4	493.5	356.4	227.4	1.85	*(1.82,1.88)*	265.9	2.19	*(2.181,2.190)*
	**2003**		251.6	293.3	324.0	381.4	469.0	336.3	217.4	1.86	*(1.83,1.89)*	253.0	2.20	*(2.195,2.204)*
	**2004**		234.7	274.1	305.1	355.0	442.1	314.7	207.4	1.88	*(1.85,1.92)*	239.5	2.22	*(2.216,2.225)*
	**2005**		217.5	250.0	284.4	331.2	412.6	291.5	195.1	1.90	*(1.86,1.93)*	227.6	2.27	*(2.264,2.275)*
	**2006**		202.1	232.1	263.6	310.8	391.7	272.2	189.5	1.94	*(1.90,1.97)*	220.4	2.34	*(2.339,2.350)*
**Overall % fall**			−65.0	−64.1	−62.5	−60.1	−55.4	−62.2	−36.9	80.3	*(76.1,84.5)*	−39.8	96.2	*(95.30,97.10)*
**Average annual % change (AAPC)** 2			−4.3	−4.2	−4.0	−3.8	−3.3	−4.0						
*95% CI for AAPC*			*(*−*4.1,* −*4.5)*	*(*−*4.0,* −*4.4)*	*(*−*3.7,* −*4.2)*	*(*−*3.5,* −*4.0)*	*(*−*3.2,* −*3.5)*	(−3.8 −4.1)						
**WOMEN**		**1982**	250.2	284.1	303.8	340.4	410.9	320.7	160.7	1.64	*(1.62,1.67)*	189.0	1.84	*(1.838,1.845)*
		**1983**	247.0	282.9	304.8	342.9	414.6	321.3	167.6	1.68	*(1.65,1.70)*	197.4	1.90	*(1.891,1.898)*
		**1984**	249.1	286.0	309.2	350.1	419.7	325.5	170.6	1.68	*(1.66,1.71)*	202.1	1.91	*(1.905,1.912)*
		**1985**	247.3	283.8	306.8	349.5	417.9	323.3	170.6	1.69	*(1.67,1.72)*	202.5	1.92	*(1.917,1.924)*
		**1986**	242.0	276.3	301.5	342.5	411.9	316.7	170.0	1.70	*(1.68,1.73)*	201.8	1.94	*(1.940,1.947)*
		**1987**	233.5	266.5	291.5	330.5	402.8	306.4	169.3	1.73	*(1.70,1.75)*	199.7	1.98	*(1.972,1.980)*
		**1988**	226.4	258.1	283.7	322.2	397.1	298.6	170.7	1.75	*(1.73,1.78)*	200.6	2.02	*(2.019,2.026)*
		**1989**	221.8	252.8	275.8	315.5	389.2	291.7	167.4	1.75	*(1.73,1.78)*	196.1	2.02	*(2.019,2.027)*
		**1990**	218.2	249.8	272.3	313.0	383.7	287.8	165.5	1.76	*(1.73,1.79)*	194.2	2.03	*(2.025,2.033)*
		**1991**	214.6	247.1	268.3	307.2	376.7	282.9	162.1	1.76	*(1.73,1.78)*	188.9	2.01	*(2.009,2.017)*
		**1992**	212.5	244.8	265.3	306.1	370.4	279.7	157.9	1.74	*(1.72,1.77)*	185.3	2.00	*(1.997,2.005)*
		**1993**	206.3	234.6	254.6	294.0	354.6	268.3	148.2	1.72	*(1.69,1.74)*	174.5	1.97	*(1.969,1.977)*
		**1994**	198.1	224.6	244.3	283.4	338.3	257.0	140.3	1.71	*(1.68,1.73)*	166.3	1.97	*(1.961,1.969)*
		**1995**	187.0	212.7	232.6	267.9	321.2	243.3	134.3	1.72	*(1.69,1.75)*	158.5	1.97	*(1.970,1.978)*
		**1996**	178.1	202.2	222.8	256.3	308.2	232.3	130.1	1.73	*(1.70,1.76)*	153.8	2.00	*(1.993,2.001)*
		**1997**	169.2	192.8	214.5	247.2	296.0	222.5	126.8	1.75	*(1.72,1.78)*	150.7	2.03	*(2.028,2.036)*
		**1998**	159.2	181.1	203.5	234.7	282.8	210.6	123.6	1.78	*(1.75,1.81)*	147.0	2.08	*(2.075,2.084)*
		**1999**	149.2	170.7	192.6	222.5	267.5	198.7	118.2	1.79	*(1.76,1.82)*	140.8	2.10	*(2.009,2.109)*
		**2000**	141.2	159.1	181.6	208.6	254.1	186.9	112.9	1.80	*(1.77,1.83)*	134.0	2.12	*(2.119,2.129)*
		**2001**	135.0	151.6	171.3	199.7	243.6	177.9	108.6	1.80	*(1.77,1.84)*	128.9	2.14	*(2.135,2.145)*
		**2002**	129.1	146.0	164.3	193.9	237.6	171.6	108.6	1.84	*(1.81,1.87)*	128.4	2.20	*(2.194,2.205)*
		**2003**	121.5	138.6	155.1	183.3	226.5	162.2	105.0	1.86	*(1.83,1.90)*	123.1	2.22	*(2.219,2.230)*
		**2004**	113.3	129.5	146.2	171.0	212.4	151.6	99.1	1.87	*(1.84,1.91)*	115.7	2.23	*(2.228,2.240)*
		**2005**	103.6	119.1	134.3	157.4	195.4	139.0	91.8	1.89	*(1.85,1.92)*	107.1	2.25	*(2.244,2.256)*
		**2006**	96.4	110.0	124.2	147.3	182.9	129.2	86.6	1.90	*(1.86,1.94)*	101.3	2.29	*(2.279,2.292)*
**Overall % fall**			−61.5	−61.3	−59.1	−56.7	−55.5	−59.7	−46.1	39.8	*(35.9,43.8)*	−46.4	52.6	*(51.80,53.50)*
**Average annual % change (AAPC)** 2			−3.9	−3.9	−3.7	−3.4	−3.3	−3.7						
*95% CI for AAPC*			*(*−*3.6,4.2)*	*(*−*3.5,* −*4.2)*	*(*−*3.3,* −*4.0)*	*(*−*3.1,* −*3.8)*	*(*−*3.2,* −*3.5)*	(−3.4, −4.0)						

Notes: Age standardised to European reference population. The rates are three-year moving averages with the central year quoted.

Standard errors and confidence intervals of age-adjusted rates available on request.

1 Relative Index of Inequality measure used is the Kunst-Mackenbach Index (KMI) derived from the HD*Calc (SEER programme). It is a regression-based relative inequality ratio between the estimated health of the person at the bottom of the socioeconomic distribution to the estimated health of the person at the top of the distribution.

2 Average annual percentage change (AAPC) is derived from the Joinpoint analysis programme. It is a weighted average of the annual % change over all period segments.

A formal test for trend in the change in rate ratios was significant (p<0.0001) for men and women.

**Figure 1:**
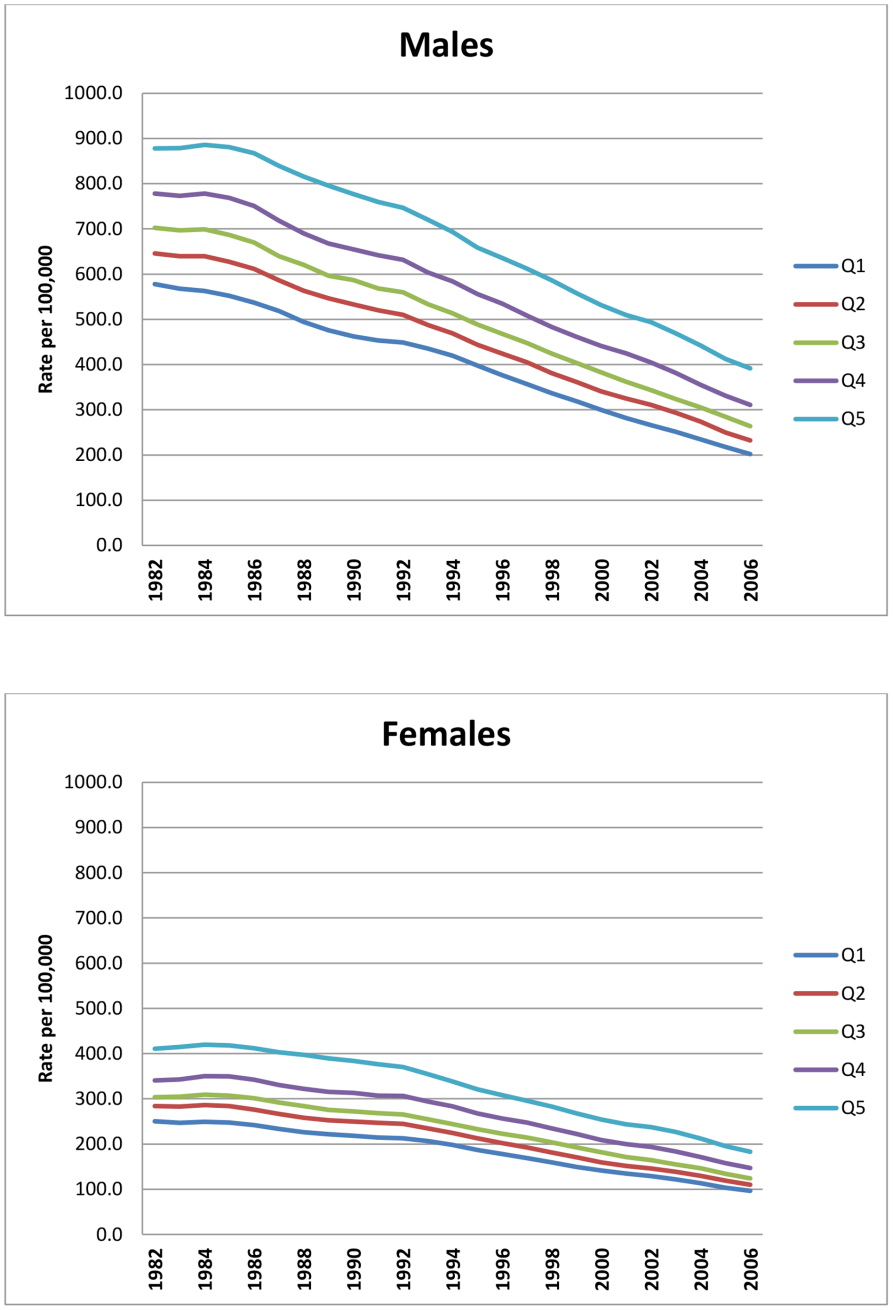
Age standardised CHD mortality rates by deprivation quintile, England 1982–2006.

The rapid decline in age-adjusted CHD mortality rates was observed in all deprivation groups. The annual average rate of decline for England was slowest in the 1980s (2.2% per year for men and 1.3% per year for women); gathered pace to 4.2% per year in the 1990s for both men and women; and accelerating even faster to 5.1% per year in the 2000s for both sexes (Table 1). Thus, the absolute gap in age-adjusted death rates between the most and least deprived groups fell by two-thirds for men (from 300 per 100,000 in 1982 to 190 per 100,000 in 2006) and almost halved for women (from 161 to 87 per 100,000, respectively) (Figure 1).

However, the narrowing of the absolute inequality gap was accompanied by a significant widening in the rate ratio between the most and least deprived groups. The widening of the rate ratios was not just an artefact of the arithmetic property of rate ratios as levels fall; despite much lower mortality levels, the annual pace of fall in the least deprived areas was the fastest. Rates fell more slowly for men and women living in the most deprived areas (3.3% per year) compared with the fall in rates observed in the least deprived areas (men 4.3% and women 3.9% per year) (Table 1).

Given the differential pace of decline, the rate ratio for men consequently rose by about 80% from 1.52 (*1.50, 1.54*) to 1.94 (*1.90, 1.97*); and rose for women too, but by half of that (40%) for men, from 1.64 (*1.62, 1.67*) to 1.90 (*1.86, 1.94*). Thus, over the quarter century, absolute inequality in CHD mortality declined but relative inequality increased significantly.

### Age-specific Changes in CHD Mortality Rates

**Figure 2:**
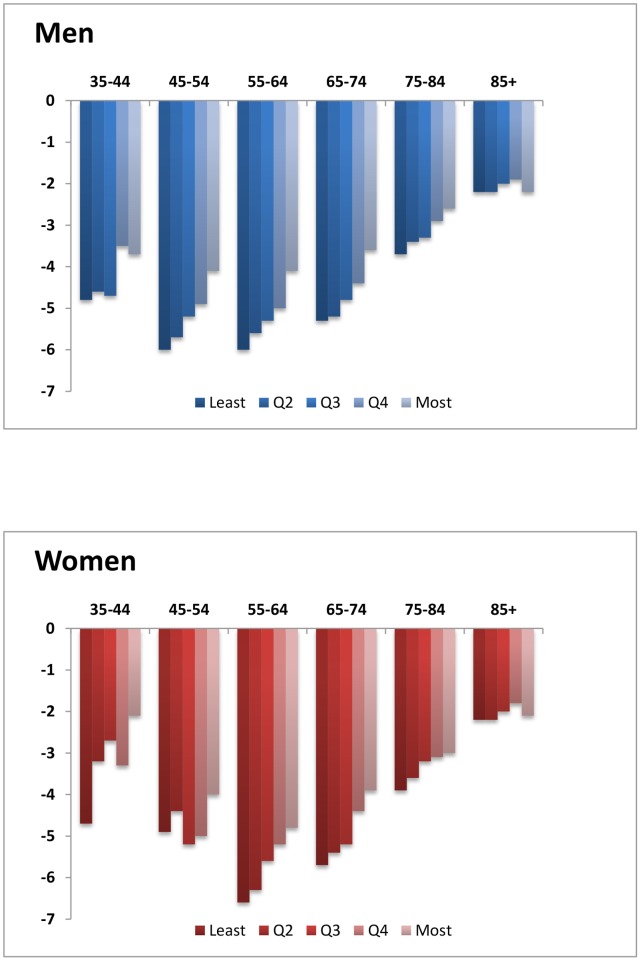
Average annual percentage change in CHD mortality rates by age group and deprivation quintile, England 1982–2006.

CHD mortality rates fell for all age groups and across all deprivation quintiles between 1982 and 2006. Absolute inequalities therefore narrowed in each age and sex group (Figure 2). However, relative inequalities widened over the same period because death rates fell differentially.

**Figure 3:**
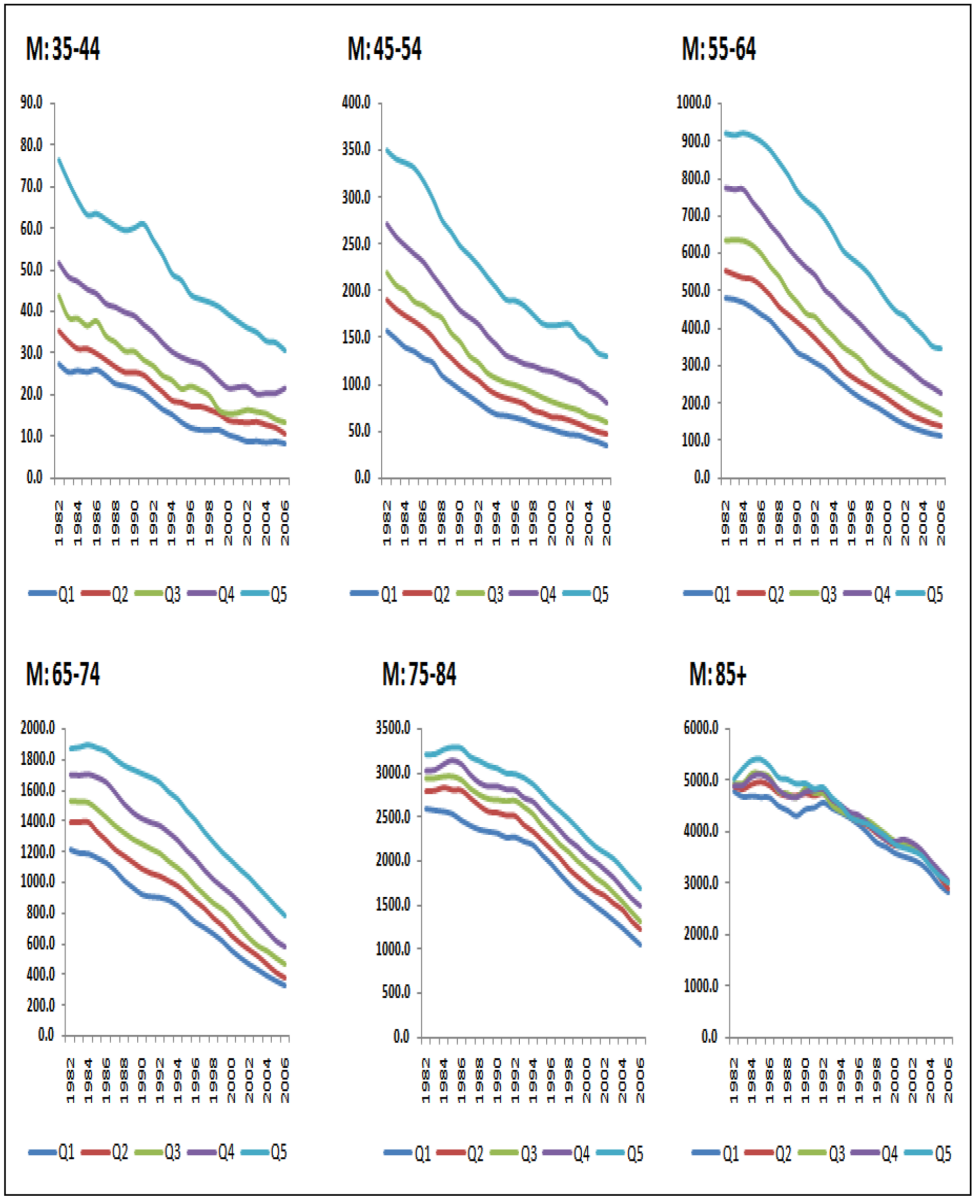
CHD mortality rates per 100,000 by age group and deprivation quintile: men, England 1982–2006.

**Figure 4:**
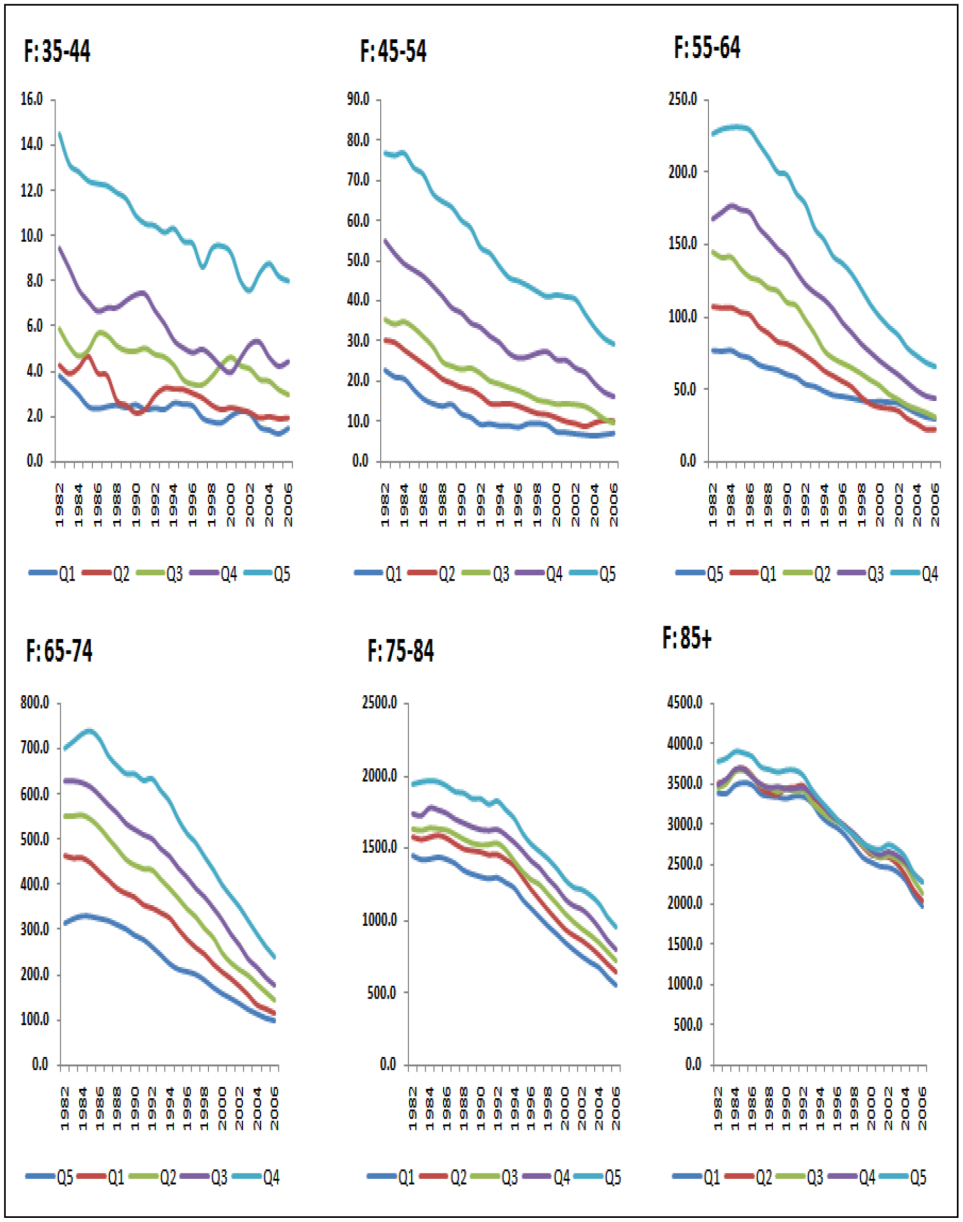
CHD mortality rates per 100,000 by age group and deprivation quintile: women, England 1982–2006.

**Figure 5:**
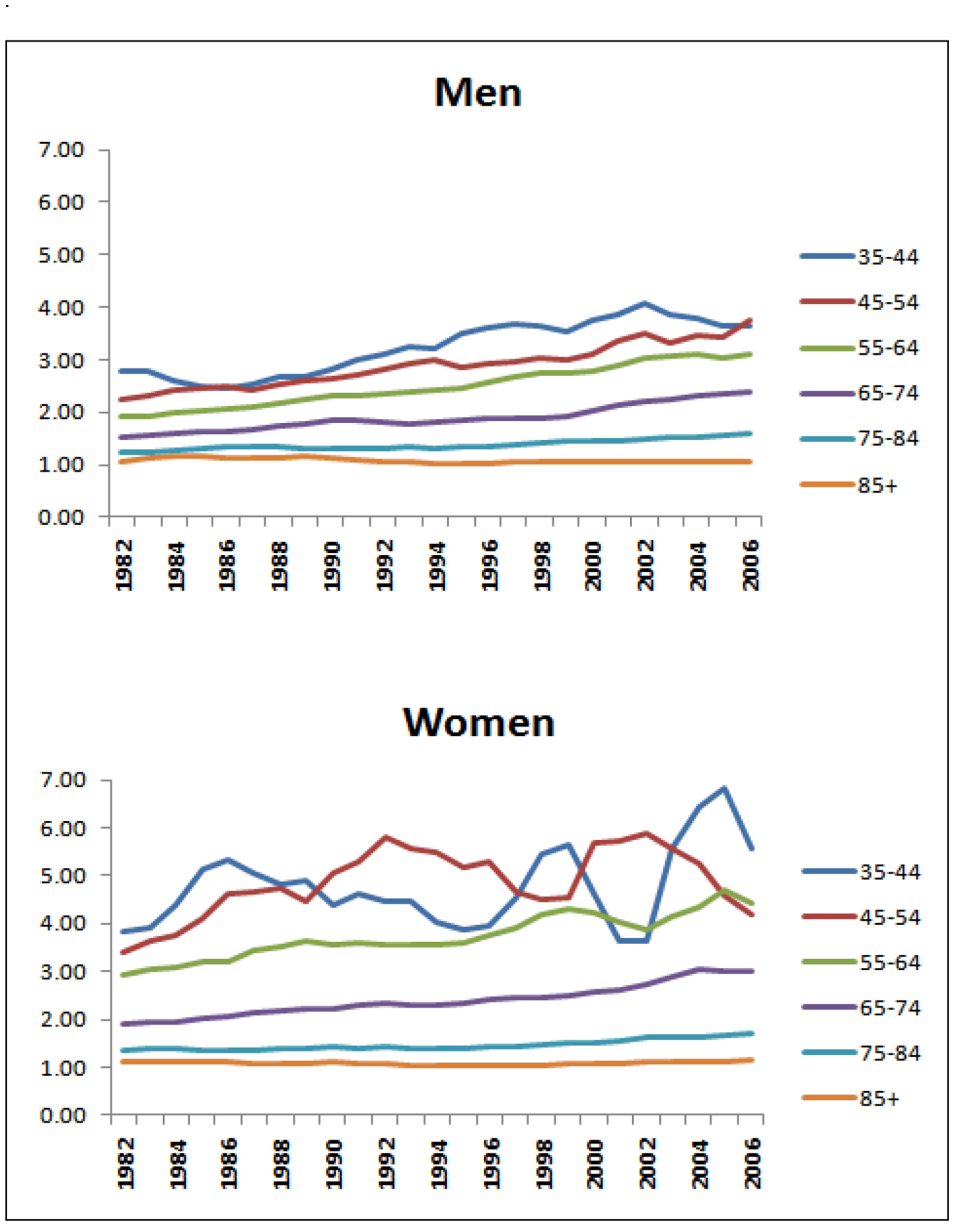
Age-specific trends in CHD mortality rate ratios between most and least deprived quintiles, England 1982–2006.

In 2006 there was a four-fold difference in rate ratios for men and a six-fold difference for women aged 35–44 (Figures 3 and 4). The rate ratio was largest for the youngest age groups and became successively shallower for older ages until by age 85 and over it stood at just a little over one, signifying only a small mortality disadvantage in the most deprived groups relative to the least deprived. Not only were the CHD mortality rate ratios larger in younger ages, they also widened more over time (Figure 5). This was more clearly visible for men because rates for young women were particularly low in the least deprived areas (under 10 per 100,000 women from 1990 onwards in the age groups 35–44 and 45–54), and therefore subject to fluctuations from one year to the next. Rate ratios for all ages drifted upwards over time because of the social gradient in the pace of decline in age-specific rates. Rates fell further in the more advantaged areas than in the deprived areas.

The average annual rate of fall over the period was larger for men than women aged up to 54, but was higher for women than men aged 55 to 84 converging finally at age 85 and over (Figure 2). The social gradient in the rate of fall averaged over the whole 25-year period of the study (or AAPCs) was most marked between ages 55–84 where the majority of CHD deaths occur (Figure 3). Among those aged 85 and over, rates of fall were modest (about 2% per annum) and the pace of fall did not vary significantly between deprivation quintiles (Figure 2). As a result, the rate ratios for those aged 85 and over stood at just over one throughout the whole period despite the rate difference in this age group narrowing by about a fifth (Figure 5).

### Time Trend Analysis in Men and Women

Partitioning the overall average percentage rate of change into time period segments showed that the annual rate of decline in CHD mortality in men for England was steeper in the most recent period from about 2000 onwards than in any of the previous periods for most age bands above 45 years (Tables 2 and 3). The exact year when the rate of decline accelerated varied across different ages. In contrast, for men aged 35–44 the overall rate of decline for England slowed significantly from −4.7% per year *(*−*5.9% to* −*3.6%*) in 1994–2000 to −2.9% per year *(*−*3.9% to* −*2.0%*) in 2000–2006.

**Table 2:** CHD mortality trends by age-group and deprivation quintiles, men, England 1982–2006.

			By deprivation quintile									
Age Group	England		Q1 (least deprived)		Q2		Q3		Q4		Q5 (most deprived)	
MEN	Period	APC (95% CI)	Period	APC (95% CI)	Period	APC (95% CI)	Period	APC (95% CI)	Period	APC (95% CI)	Period	APC (95% CI)
**35–44:**	1982–1991	−3.4* (−3.7, −3.0)	1982–1986	−1.1 (−3.6,1.4)	1982–1991	−3.9* (−4.5, −3.2)	1982–1997	−4.7* (−5.1, −4.2)	1982–1990	−3.3* (−3.8, −2.8)	1982–1985	−6.2* (−7.7, −4.6)
	1991–1994	−6.5* (−11.0, −1.7)	1986–1991	−4.4* (−6.8, −1.9)	1991–1994	−7.9 (−15.6,0.5)	1997–2000	−9.6 (−20.9,3.3)	1990–2000	−5.2* (−5.7, −4.7)	1985–1991	−0.8 (−1.5,0.0)
	1994–2000	−4.7* (−5.9, −3.6)	1991–1996	−9.5* (−12.3, −6.6)	1994–2006	−4.3* (−4.9, −3.7)	2000–2003	1.9 (−11.1,16.8)	2000–2004	−2.7 (−5.7,0.4)	1991–1995	−6.0* (−7.8, −4.2)
	2000–2006	−2.9* (−3.9, −2.0)	1996–2006	−4.0* (−4.9, −3.1)			2003–2006	−6.4 (−12.8,0.5)	2004–2006	3.1 (−3.0,9.6)	1995–2006	−3.7* (−4.0, −3.4)
**AAPC**	1982–2006	−4.0* (−4.6, −3.3)		−4.8* (−5.7, −3.9)		−4.6* (−5.6, −3.6)		−4.7* (−6.9, −2.5)		−3.5* (−4.2, −2.8)		−3.7* (−4.1, −3.3)
												
**45–54:**	1982–1986	−3.7* (−4.4, −3.1)	1982–1987	−4.9* (−5.6, −4.2)	1982–1986	−4.1* (−5.0, −3.2)	1982–1988	−4.0* (−4.5, −3.5)	1982–1986	−3.9* (−4.7, −3.1)	1982–1985	−1.8 (−3.9,0.4)
	1986–1994	−6.7* (−7, −6.4)	1987–1993	−8.0* (−8.8, −7.2)	1986–1994	−7.1* (−7.5, −6.6)	1988–1993	−7.8* (−8.8, −6.8)	1986–1996	−5.8* (−6.1, −5.5)	1985–1995	−5.3* (−5.8, −4.9)
	1994–2003	−3.8* (−4.1, −3.5)	1993–2003	−4.7* (−5.1, −4.3)	1994–2002	−4.7* (−5.2, −4.2)	1993–2003	−4.3* (−4.6, −4.0)	1996–2003	−2.9* (−3.5, −2.2)	1995–2003	−2.7* (−3.5, −1.9)
	2003–2006	−6.1* (−7.6, −4.6)	2003–2006	−7.9* (−10.4, −5.2)	2002–2006	−6.4* (−7.8, −5.0)	2003–2006	−6.0* (−8.1, −3.8)	2003–2006	−7.7* (−9.7, −5.7)	2003–2006	−5.7* (−8.8, −2.4)
**AAPC**	1982–2006	−5.1* (−5.3, −4.8)		−6.0* (−6.4, −5.6)		−5.7* (−6.0, −5.3)		−5.2* (−5.5, −4.8)		−4.9* (−5.2, −4.6)		−4.1* (−4.6, −3.5)
												
**55–64:**	1982–1985	−0.8 (−1.6,0.0)	1982–1985	−1.4 (−3.0,0.2)	1982–1985	−1.1 (−2.5,0.3)	1982–1985	−0.5 (−1.6,0.7)	1982–1984	−0.1 (−1.0,0.9)	1982–1986	−0.4 (−1.1,0.3)
	1985–1993	−4.9* (−5.1, −4.6)	1985–1994	−5.7* (−6.1, −5.3)	1985–1991	−4.8* (−5.4, −4.1)	1985–1994	−5.4* (−5.7, −5.2)	1984–1992	−4.4* (−4.6, −4.3)	1986–1993	−3.9* (−4.3, −3.5)
	1993–2006	−6.5* (−6.6, −6.4)	1994–2004	−7.6* (−8.0, −7.2)	1991–2006	−6.8* (−7.0, −6.7)	1994–2006	−6.4* (−6.6, −6.2)	1992–1997	−5.2* (−5.6, −4.8)	1993–1998	−4.8* (−5.8, −3.9)
			2004–2006	−4.7 (−9.9,0.7)					1997–2006	−6.4* (−6.5, −6.2)	1998–2006	−5.6* (−6.0, −5.2)
**AAPC**	1982–2006	−5.3* (−5.4, −5.1)		−5.9* (−6.4, −5.4)		−5.6* (−5.9, −5.4)		−5.3* (−5.5, −5.1)		−5.0* (−5.1, −4.9)		−4.1* (−4.4, −3.8)
**65–74:**	1982–1984	0.0 (−2.0,1.9)	1982–1984	−1.1 (−5.9,3.9)	1982–1984	−0.9 (−3.6,1.8)	1982–1984	−0.5 (−2.5,1.6)	1982–1985	−0.5 (−1.9,1.0)	1982–1984	1.0 (−0.1,2.1)
	1984−1994	−3.0* (−3.1, −2.8)	1984–1994	−3.5* (−3.9, −3.0)	1984–1996	−3.5* (−3.7, −3.3)	1984–1994	−3.1* (−3.3, −2.9)	1985–1994	−3.0* (−3.4, −2.7)	1984–1993	−1.8* (−1.9, −1.7)
	1994–2000	−5.7* (−6.2, −5.2)	1994–1998	−5.4* (−8.1, −2.6)	1996–2003	−7.4* (−7.9, −6.8)	1994–1999	−5.6* (−6.4, −4.9)	1994–2001	−5.2* (−5.8, −4.6)	1993–2002	−4.8* (−5.0, −4.7)
	2000–2006	−7.8* (−8.3, −7.4)	1998–2006	−8.5* (−9.2, −7.8)	2003–2006	−10.1* (−12.1, −8.1)	1999–2006	−7.7* (−8.0, −7.3)	2001–2006	−7.8* (−8.8, −6.8)	2002–2006	−6.7* (−7.2, −6.1)
***AAPC***	1982–2006	−4.7* (−4.9, −4.4)		−5.3* (−5.9, −4.7)		−5.3* (−5.6, −4.9)		−4.8* (−5.0, −4.5)		−4.4* (−4.7, −4.1)		−3.5* (−3.7, −3.4)
**75–84:**	1982–1985	0.2 (−1.0,1.3)	1982–1994	−1.5* (−1.6, −1.3)	1982–1984	1.1 (−2.1,4.4)	1982–1984	0.7 (−2.6,4.0)	1982–1985	0.9 (−0.7,2.5)	1982–1985	0.9* (0.0,1.9)
	1985–1994	−1.7* (−1.9, −1.5)	1994–2003	−5.3* (−5.5, −5.0)	1984–1993	−1.7* (−2.0, −1.4)	1984–1994	−1.5* (−1.8, −1.3)	1985–1994	−1.7* (−2.0, −1.3)	1985–1994	−1.5* (−1.7, −1.3)
	1994–2003	−4.6* (−4.9, −4.3)	2003–2006	−7.4* (−8.6, −6.1)	1993–2004	−4.6* (−4.9, −4.4)	1994–2003	−4.7* (−5.1, −4.4)	1994–2003	−4.2* (−4.5, −3.8)	1994–2003	−3.9* (−4.1, −3.7)
	2003–2006	−6.9* (−8.2, −5.6)			2004–2006	−8.2* (−11.6, −4.7)	2003–2006	−7.3* (−9.1, −5.4)	2003–2006	−6.5* (−8.3, −4.7)	2003–2006	−5.7* (−6.8, −4.6)
***AAPC***	1982–2006	−3.2* (−3.5, −3.0)		−3.7* (−3.9, −3.5)		−3.4* (−3.8, −3.0)		−3.3* (−3.7, −2.9)		−2.9* (−3.2, −2.6)		−2.6* (−2.8, −2.4)
												
**85+**	1982–1992	−0.7* (−1.1, −0.3)	1982–1989	−1.4* (−2.0, −0.9)	1982–1992	−0.4* (−0.7, −0.1)	1982–1984	1.7 (−4.3,8.0)	1982–1992	−0.5 (−0.9,0.0)	1982–1984	3.6 (−0.4,7.8)
	1992–2003	−2.5* (−2.8, −2.2)	1989–1992	1.7 (−1.9,5.4)	1992–2000	−2.8* (−3.2, −2.4)	1984–1992	−1.1* (−1.8, −0.4)	1992–1999	−2.9* (−3.8, −2.0)	1984–1992	−1.6* (−2.1, −1.1)
	2003–2006	−5.7* (−7.7, −3.7)	1992–2003	−2.9* (−3.1, −2.6)	2000–2003	−0.9 (−3.8,2.2)	1992–2003	−2.3* (−2.7, −1.9)	1999–2002	−0.7 (−5.8,4.7)	1992–2003	−2.8* (−3.0, −2.5)
			2003–2006	−5.5* (−7.0, −4.0)	2003–2006	−7.5* (−8.9, −6.1)	2003–2006	−5.7* (−7.9, −3.3)	2002–2006	−5.3* (−6.9, −3.7)	2003–2006	−5.1* (−6.7, −3.4)
***AAPC***	1982–2006	−2.2* (−2.5, −1.9)		−2.2* (−2.7, −1.8)		−2.2* (−2.6, −1.8)		−2.0* (−2.6, −1.4)		−2.0* (−2.7, −1.3)		−2.2* (−2.5, −1.8)

Notes:

APC = annual percentage change (for each period segment).

AAPC = annual average percentage change (weighted average of annual percentage changes over all period segments).

‘*’ Indicates statistically significant change compared to no change (in AAPC) or relative to the previous segment (in APC).

**Table 3:** CHD mortality trends by age-group and deprivation quintiles, women, England 1982–2006.

			By deprivation quintile									
Age Group	England		Q1 (least deprived)		Q2		Q3		Q4		Q5 (most deprived)	
WOMEN	Period	APC (95% CI)	Period	APC (95% CI)	Period	APC (95% CI)	Period	APC (95% CI)	Period	APC (95% CI)	Period	APC (95% CI)
35–44:	1982–1984	−7.9* (−13.6, −1.7)	1982–1985	−12.7 (−24.2,0.4)	1982–1985	4.7 (−2.9,13.0)	1982–1993	−1.2* (−2.4,0.0)	1982–1986	−8.4* (−13.9, −2.6)	1982–2002	−2.5* (−2.8, −2.2)
	1984–2006	−2.4* (−2.5, −2.2)	1985–2001	−1.4* (−2.7,0.0)	1985–1990	−14.0* (−18.5, −9.4)	1993–1996	−12.0 (−27.9,7.3)	1986–1991	2.7 (−3.7,9.5)	2002–2006	0.2 (−3.5,3.9)
			2001–2006	−9.4* (−16.3, −1.9)	1990–1993	15.7 (−3.6,38.9)	1996–2000	9.1 (−1.0,20.4)	1991–1995	−10.4 (−19.7,0.1)		
					1993–2006	−4.5* (−5.4, −3.6)	2000–2006	−7.0* (−10.0, −3.9)	1995–2006	−0.6 (−2.1,1.0)		
***AAPC***	***1982–2006***	−2.8* (−***3.3,*** −***2.3)***		−4.6* (−***6.8,*** −***2.3)***		−3.2* (−***5.6,*** −***0.7)***		−***2.5 (***−***5.2,0.4)***		−2.9* (−***5.2,*** −***0.6)***		−2.1* (−***2.7,*** −***1.5)***
												
45–54:	1982–1984	−2.8* (−5.0, −0.5)	1982–1994	−7.9* (−8.7, −7.1)	1982–1993	−6.3* (−6.7, −5.9)	1982–1985	−1.1 (−5.0,2.9)	1982–1996	−5.2* (−5.4, −4.9)	1982–1984	0.6 (−3.5,5.0)
	1984–1995	−5.8* (−6.0, −5.6)	1994–1998	2.6 (−5.5,11.3)	1993–1996	−2.9 (−8.9,3.5)	1985–1988	−9.2* (−16.8, −0.9)	1996–1999	1.0 (−5.5,7.9)	1984–1997	−4.4* (−4.7, −4.1)
	1995–2002	−2.7* (−3.2, −2.2)	1998–2002	−8.7* (−16.2, −0.6)	1996–2003	−5.7* (−6.9, −4.6)	1988–2004	−4.3* (−4.8, −3.9)	1999–2003	−5.1* (−8.3, −1.8)	1997–2002	−1.5 (−3.2,0.3)
	2002–2006	−6.2* (−7.2, −5.2)	2002–2006	1.0 (−4.9,7.2)	2003–2006	4.9* (1.0,8.9)	2004–2006	−12.1 (−22.9,0.1)	2003–2006	−10.5* (−14.0, −6.9)	2002–2006	−8.0* (−9.8, −6.2)
***AAPC***	***1982–2006***	−4.7* (−***5.0,*** −***4.5)***		−4.9* (−***6.8,*** −***2.9)***		−4.4* (−***5.3,*** −***3.5)***		−5.2* (−***6.7,*** −***3.8)***		−5.1* (−***6.1,*** −***4.1)***		−4.0* (−***4.5,*** −***3.4)***
												
55–64:	1982–1985	0.4 (−0.8,1.6)	1982–1985	−1.0 (−4.2,2.3)	1982–1991	−3.3* (−3.7, −3.0)	1982–1984	3.1* (0.5,5.8)	1982–1985	1.0 (−0.5,2.5)	1982––1985	1.6 (−0.2,3.4)
	1985–1990	−3.8* (−4.6, −3)	1985–1993	−5.0* (−6.0, −4.1)	1991–1995	−10.1* (−12.4, −7.8)	1984–1987	−2.7* (−5.1, −0.1)	1985–1990	−3.4* (−4.3, −2.4)	1985–1989	−2.2* (−4.0, −0.4)
	1990–1998	−6.6* (−7.0, −6.2)	1993–2002	−7.8* (−8.8, −6.9)	1995–1998	−4.7 (−9.9,0.9)	1987–1995	−5.5* (−5.8, −5.1)	1990–1997	−6.0* (−6.6, −5.4)	1989–1998	−5.3* (−5.8, −4.8)
	1998–2006	−8.4* (−8.9, −8.0)	2002–2006	−10.7* (−13.9, −7.4)	1998–2006	−8.4* (−9.0, −7.7)	1995–2006	−7.9* (−8.1, −7.6)	1997–2006	−7.5* (−7.9, −7.1)	1998–2006	−7.8* (−8.4, −7.2)
***AAPC***	***1982–2006***	−5.8* (−***6.1,*** −***5.5)***		−6.6* (−***7.3,*** −***5.8)***		−6.3* (−***7.1,*** −***5.6)***		−5.6* (−***5.9,*** −***5.2)***		−5.2* (−***5.5,*** −***4.9)***		−4.8* (−***5.2,*** −***4.4)***
**65–74:**	1982–1985	−0.1 (−1.1,1.0)	1982–1984	−0.7 (−3.9,2.6)	1982–1984	0.5 (−3.8,4.9)	1982–1984	0.1 (−1.6,1.9)	1982–1984	2.9 (−1.5,7.4)	1982–1985	0.8 (0.0,1.5)
	1985–1994	−2.8* (−3.1, −2.6)	1984–1994	−3.4* (−3.7, −3.1)	1984–1994	−3.4* (−3.8, −3.0)	1984–1994	−3.0* (−3.2, −2.8)	1984–1994	−2.3* (−2.7, −1.9)	1985–1993	−1.6* (−1.8, −1.4)
	1994–2000	−6.3* (−6.9, −5.8)	1994–2001	−7.2* (−7.8, −6.5)	1994–1998	−6.2* (−8.7, −3.6)	1994–1999	−5.4* (−6.1, −4.7)	1994–2002	−6.2* (−6.9, −5.5)	1993–2003	−5.5* (−5.7, −5.3)
	2000–2006	−8.8* (−9.4, −8.3)	2001–2006	−10.3* (−11.5, −9.2)	1998–2006	−8.8* (−9.5, −8.0)	1999–2006	−9.3* (−9.7, −9.0)	2002–2006	−9.3* (−11.5, −7.1)	2003–2006	−8.7* (−9.9, −7.4)
***AAPC***	***1982–2006***	−4.9* (−***5.1,*** −***4.7)***		−5.8* (−***6.2,*** −***5.4)***		−5.4* (−***5.9,*** −***4.8)***		−5.1* (−***5.4,*** −***4.9)***		−4.4* (−***4.9,*** −***3.9)***		−3.9* (−***4.1,*** −***3.7)***
												
**75–84:**	1982–1984	0.5 (−1.9,2.9)	1982–1985	−0.1 (−1.4,1.3)	1982–1985	0.0 (−1.5,1.5)	1982–1985	0.0 (−1.7,1.7)	1982–1984	1.4 (−1.4,4.2)	1982–1984	0.8 (−2.2,3.9)
	1984–1993	−1.2* (−1.4, −0.9)	1985–1994	−1.7* (−2.0, −1.4)	1985–1993	−1.2* (−1.5, −0.8)	1985–1993	−1.1* (−1.5, −0.6)	1984–1994	−1.3* (−1.5, −1.1)	1984–1993	−1.3* (−1.6, −0.9)
	1993–2004	−5.0* (−5.2, −4.8)	1994–2004	−5.8* (−6.1, −5.6)	1993–2004	−5.6* (−5.9, −5.4)	1993–2003	−4.9* (−5.2, −4.5)	1994–2003	−4.6* (−4.9, −4.3)	1993–2004	−4.2* (−4.5, −3.9)
	2004–2006	−8.7* (−11.7, −5.5)	2004–2006	−8.8* (−12.0, −5.4)	2004–2006	−8.0* (−11.8, −4.1)	2003–2006	−7.0* (−9.2, −4.8)	2003–2006	−7.5* (−9.3, −5.7)	2004–2006	−7.0* (−11.2, −2.6)
***AAPC***	***1982–2006***	−3.4* (−***3.8,*** −***3.1)***		−3.9* (−***4.2,*** −***3.5)***		−3.7* (−***4.0,*** −***3.3)***		−3.3* (−***3.7,*** −***2.9)***		−3.1* (−***3.5,*** −***2.8)***		−2.9* (−***3.4,*** −***2.5)***
												
**85+**	1982–1992	−0.5* (−0.9, −0.2)	1982–1992	−0.3 (−0.7,0.1)	1982–1992	−0.5* (−1.0,0.0)	1982–1984	3.0 (−2.2,8.5)	1982–1993	−0.6* (−0.9, −0.3)	1982–1992	−0.8* (−1.1, −0.4)
	1992–2000	−3.3* (−3.9, −2.7)	1992–2000	−3.4* (−4.0, −2.9)	1992–2004	−3.0* (−3.4, −2.6)	1984–1992	−1.0* (−1.7, −0.4)	1993–2000	−3.4* (−4.2, −2.6)	1992–1999	−3.9* (−4.5, −3.2)
	2000–2003	−0.8 (−5.4,4.0)	2000–2003	−1.4 (−5.5,2.9)	2004–2006	−7.5* (−13.4, −1.3)	1992–2004	−2.6* (−2.9, −2.3)	2000–2003	−0.6 (−5.4,4.5)	1999–2003	−0.2 (−2.3,2.0)
	2003–2006	−5.9* (−8.2, −3.5)	2003–2006	−6.4* (−8.5, −4.3)			2004–2006	−6.4* (−10.9, −1.7)	2003–2006	−4.6* (−7.0, −2.1)	2003–2006	−5.6* (−7.8, −3.4)
***AAPC***	***1982–2006***	−2.2* (−***2.8,*** −***1.5)***		−2.3* (−***2.8,*** −***1.7)***		−2.3* (−***2.9,*** −***1.8)***		−2.0* (−***2.5,*** −***1.4)***		−1.9* (−***2.6,*** −***1.2)***		−2.2* (−***2.7,*** −***1.7)***

Average annual percentage change (AAPC) and annual percentage change (APC) per segment calculated using Joinpoint software analysis.

Notes:

APC = annual percentage change (for each period segment).

AAPC = annual average percentage change (weighted average of annual percentage changes over all period segments).

‘*’ Indicates statistically significant change compared to no change (in AAPC) or relative to the previous segment (in APC).

The average annual percentage decline in rates for women exhibited a similar time pattern of accelerated falls in the 2000s in all ages above 45 (Table 3). Rates for the youngest age band (35–44) were very unstable with no clear trend detectable in the most recent period (reflecting small numbers of events). In the next higher age band (age 45–54), the overall pace of fall doubled in the most recent segment (from - 2.7% *(*−*3.2% to* −*2.2%*) in 1995–2002 to −6.2%, *(*−*7.2% to* −*5.2%*) in 2002–2006. Rates of decline for older ages were all significantly higher in the most recent period than in any period previously.

### Time Trend Analysis – by Deprivation Quintiles and Sex

Examination of deprivation-specific age trends revealed a similar slowing in the rate of fall in younger men aged less than 45 in all quintiles except the third quintile (Table 2). In contrast, the pace of fall in young women aged 45–54 in the two least deprived quintiles flattened substantially (to 1.0% in quintile one, not significantly different from 0% at the 95% confidence level) and actually reversed for quintile two with rates *rising* by about 5% per year between 2003–2006 (+4.9%,+*1.0% to +8.9%*) (Table 3).

From age 55 onwards, the pace of fall in the most recent period was significantly more rapid than in the preceding period in virtually all age groups and across all deprivation quintiles. Furthermore, for each age group and across sexes, rates of fall were invariably lower in the most deprived than the least deprived quintile groups.

## Discussion

### Summary of Study Findings

This study investigated trends in the socio-economic patterning of CHD mortality between 1982 and 2006 in the English population aged 35 and older. CHD death rates fell dramatically, by approximately 60% in men and women. Rates of fall accelerated from 2000s onwards, with the steepest falls in the most advantaged group. Hence, substantial social inequalities in CHD mortality persisted in England throughout the period. For ages less than 45 years, CHD death rates were up to six times higher in deprived compared to advantaged groups. Encouragingly, the gap between the most and least deprived quintiles closed year on year, such that by 2006 absolute inequalities were approximately half those in 1982. However, because the pace of fall was steeper in the least deprived areas, relative inequalities widened over the same period such that by 2006, the ratio of age-adjusted CHD death rates was about twice as high.

Examining age-specific trends reveals two contrasting patterns from about 2000: the secular fall in CHD mortality stalled for ages 35–54 across all socioeconomic groups, but accelerated for middle aged and elderly men and women. The latter were socially graded, being slowest for the most deprived groups. As most CHD deaths occur at older ages, the net effect of these contrasting trend patterns on the overall age-standardised rate was one of significantly accelerated fall in the early years of the 21^st^ century for both sexes and across all deprivation quintiles, but with relative inequalities widening.

### Comparisons with Other Studies

This study confirms previous reports of a slowing of the pace of decline in CHD mortality trends in young adults in England [5], [19]. First, the flattening in the younger men (35–44) clearly continues (even after adding a few crucial years in early 2000s to update the time series). The pattern of change for men and women aged 45–54 is slightly different. In previous analysis for England and Wales, this age group experienced a slowing down in the pace of decline, starting in the mid-1990s [5]. In this updated analysis, the flattening is confirmed for the period 1994/5-2002, but thereafter instead of stagnating, rates began to fall strongly again. This intriguing pattern has also recently been observed in the Netherlands for men and women aged 35–54 [20].

A recent analysis of the WHO Health For All database, focusing on age-standardised rates for adults aged 35–44, confirmed the flattening periods in Scotland and the “speed up” observed in the Netherlands in the early 2000s, but did not comment on flattening in England [21]. Although they looked at a similar time span, they compared change across three fixed time points, each a decade apart. Our findings therefore highlight the added value of using more granular data to pinpoint turning points more precisely, and also the need to frequently update trend analysis as new information becomes available.

The flattening in CHD mortality trends in younger men and women in England was relatively uniform across social quintiles. This offers an intriguing contrast with Scotland where it was limited to young adults living in deprived areas [14].

Socioeconomic differentials in the pace of change in age-adjusted rates have been reported in other settings, but often without examining age specific rates. Marked social differences, with widening of relative inequalities in premature coronary heart disease mortality rates at the turn of the century have been described in Great Britain [22] and in six European countries [23]. In the US, slower decline in CHD and stroke in the least educated was observed, particularly in African Americans with low educational attainment [24]. In New Zealand, a similar slowing of decline in CHD mortality rates has been described for Maoris and Pacific Islanders, considered more deprived than Europeans [8].

### Strengths and Limitations

To our knowledge, this is the first study to uncover the socioeconomic dimension in the conflicting pattern of change in age-specific CHD mortality rates at the start of the 21^st^ century in England. Strengths of our study include: the use of data on the entire population rather than a potentially unrepresentative sample; the analysis of year-on-year change, rather than selected atypical endpoint years, to uncover the underlying trends over a quarter century; and the use of trend regression analysis software (Joinpoint) to pinpoint turning points.

The Joinpoint regression analysis is able to identify periods of similar annual percent changes; avoiding the need to pre-specify time periods (which may then bias the way in which the trends are analysed). Moreover, because the maximum numbers of possible join points was deliberately limited in this study, each segment within the overall trend was based on more data points and therefore better captured the true underlying shifts in the pace of CHD mortality change over the quarter century, undistorted by short-run variability in mortality rates. Furthermore, this population is large enough to allow the calculation of relatively robust age- and sex-specific estimates by deprivation quintiles. However, a disadvantage of Joinpoint analysis is that the turning points, and hence the associated time intervals, do not coincide for each population subgroup making it more difficult to identify potential drivers and thereby inform policy action.

This study also has several limitations. Cause of death coding is prone to misclassification and inconsistencies, particularly with changes over time in classification systems and coding rules. Results of necropsy studies have also found that the accuracy of death certification varies by type of disease and patients’ age [25]. For CHD deaths, however, misclassification and inaccurate certification were relatively minor issues as evidenced by studies using dual ICD coding methods to assess the impact of major classification change [26] and morbidity-mortality linked data to identify inconsistencies in cause-coding [25]. Misclassification of cause of death recording (and variation in such misclassification over the study period) would need to be differential across deprivation quintiles to bias our estimates of CHD mortality inequality. However, any such bias is likely to be small as there is no reason to suppose that medical certification practices were systematically and substantially related to deprivation.

We used the overall IMD score of a small area in 2007 to allocate it to a quintile group and this categorisation remained fixed over the entire period of the analysis (Text S1). This was partly for practical reasons - there was no equivalent composite score of multiple deprivation available until the late 1990s - and partly because the *relative* ranking of small areas in England has remained remarkably stable over long periods whatever measure of relative deprivation is used [27]. Selective (net) migration patterns might still hamper trend analysis of socioeconomic mortality differentials when using area-based measures of socio-economic position [28]–[29]. Our finding of widening relative social inequalities in CHD mortality is consistent with findings from cohort follow-up studies using individual social position [30]. However, this is an ecological study and we can only draw firm conclusions about trends in CHD mortality in deprived areas, not deprived individuals. The small areas on which the IMD is based are quite similar in size (c. 1500 persons, falling to c. 1000 age 25 or over), and are socially segregated, but not all socially disadvantaged people live in deprived areas, and vice-versa. But because area-based deprivation measures capture both the contextual and compositional aspects of deprivation, they may be a more reliable measure of socioeconomic inequalities than disadvantage measured between groups based on individual social position alone.

The overall IMD score is a composite of seven domains, including a health and disability domain which uses premature total mortality in its derivation. Including this domain to analyse mortality trends might have induced some circular inference. However, removing the health domain in other analyses had little effect on either the assignment of areas into specific deprivation quintiles or the relationship between area-based deprivation and health [31].

### Plausible Explanations of Persistent Inequalities and Trends

The persistence of significant relative inequalities and the slowing down of the mortality decline in younger cohorts cannot be plausibly explained by a dramatic deterioration in medical care. Indeed, the National Health Service (NHS) in England has been remarkably effective in delivering evidence-based care for coronary heart disease patients across the socioeconomic spectrum [32]–[33]. Thus, socioeconomic differences in risk factor trends, and hence CHD incidence, appear the most likely candidate to explain the social gradient in mortality patterns.

Risk factor trends in England by socioeconomic circumstance appear complex [34]. Between 1994 and 2008, the prevalence of smoking, high blood pressure and raised cholesterol decreased in most deprivation quintiles, with relative inequalities neither widening nor narrowing significantly. However, inequalities increased in obesity and diabetes and high blood pressure particularly in younger women.

Acceleration in the decline in CHD mortality among middle-aged and older people might partly reflect the impact on case-fatality of the doubling of uptake of effective drug therapies for community based patients with chronic disease, for it is they who represent the largest CHD burden [33]. On-going modelling studies to understand the evolution of social differentials in the drivers of changes in CHD incidence and changes in case-fatality in England [33] and Scotland might shed further light on this issue.

Life expectancy has increased markedly over the period of the study, particularly at older ages. It has increased for all groups, but more rapidly for the most advantaged resulting in an increase in relative inequality in life expectancy between socioeconomic groups in England and Wales [35]. The fall in CHD mortality since the 1970s has played a major role in the overall increase in life expectancy; and equally, our study shows that differentials in the pace of its fall between groups have contributed to the widening of relative inequalities in life expectancy.

### Conclusions

Coronary heart disease remains the leading cause of death in the UK, and an important contributor to socio-economic inequalities in life expectancy. Although absolute inequalities have declined over time, the widening of relative inequalities and the recent slowing of the decline in CHD mortality rates in young adults suggest that the epidemic is far from being controlled.

The NHS success in providing equitable care to CHD patients should now be matched by an equally strong emphasis on equitable prevention. Population level policies, such as tobacco legislation and dietary salt intake reduction have the potential to reduce CHD burden whilst also reducing socio-economic inequalities [36].

## Supporting Information

Table S1 **Men: CHD death counts, mortality rates per 100,000 and standard errors of rates by age group, deprivation quintile and year. 1982–2006, England.**
(XLSX)

Table S2 **Women: CHD death counts, mortality rates per 100,000 and standard errors of rates by age group, deprivation quintile and year. 1982–2006, England.**
(XLSX)

Text S1 **Implications of using a fixed IMD quintile allocation for small areas in England from 1981 to 2007.**
(DOCX)

## References

[1] Siegler V, Langford A, Johnson B (2008) Regional differences in male mortality inequalities using the National Statistics Socio-Economic Classification, England and Wales, 2001–03. Health Stat Q 40: 6–17. 19093636

[2] Langford A, Johnson B, Al-Hamad A (2009) Social inequalities in female mortality by region and by selected causes of death, England and Wales, 2001–03. Health Stat Q 44: 7–26. 10.1057/hsq.2009.35 19994749

[3] Scarborough P, Allender S, Peto V, Rayner M (2008) Regional and social differences in Coronary Heart Disease 2008. London: British Heart Foundation.

[4] The Marmot Review (2010) The social gradient in cardiovascular disease explains more than half of the mortality gap between rich and poor. *Fair Society, Healthy Lives: A Strategic Review of Health Inequalities in England Post-2010* (www.marmotreview.org).

[5] O’Flaherty M, Ford E, Allender S, Scarborough P, Capewell S (2008) Coronary heart disease trends in England and Wales from 1984 to 2004: concealed levelling of mortality rates among young adults. Heart 94: 178–181. 17641070 10.1136/hrt.2007.118323

[6] Wilson A, Siskind V (1995) Coronary heart disease mortality in Australia: is mortality starting to increase among young men? Int J Epidemiol 24: 678–684. 8550263 10.1093/ije/24.4.678

[7] O’Flaherty M, Allender S, Taylor R, Stevenson C, Peeters A, et al (2012) The decline in coronary heart disease mortality is slowing in young adults (Australia 1976–2006): a time trend analysis. Int J Cardiol 158(2): 193–198. 21288580 10.1016/j.ijcard.2011.01.016

[8] Tobias M, Sexton K, Mann S, Sharpe N (2006) How low can it go? Projecting ischaemic heart disease mortality in New Zealand to 2015. N Z Med J 119 (1232): U1932. 16633391

[9] Danaei G, Finucane MM, Lu Y, Singh GM, Cowan MJ, et al (2011) National, regional, and global trends in fasting plasma glucose and diabetes prevalence since 1980: systematic analysis of health examination surveys and epidemiological studies with 370 country-years and 2·7 million participants. Lancet 378: 31–40. 21705069 10.1016/S0140-6736(11)60679-X

[10] Finucane MM, Stevens GA, Cowan MJ, Danaei G, Lin JK, et al (2011) National, regional, and global trends in body-mass index since 1980: systematic analysis of health examination surveys and epidemiological studies with 960 country-years and 9·1 million participants. Lancet 377: 557–567. 21295846 10.1016/S0140-6736(10)62037-5 PMC4472365

[11] Capewell S, Ford ES, Croft JB, Critchley JA, Greenlund KJ, et al (2010) Cardiovascular risk factor trends and potential for reducing coronary heart disease mortality in the United States of America. Bull World Health Organ 88: 120–130. 20428369 10.2471/BLT.08.057885 PMC2814476

[12] Ford ES, Li C, Zhao G, Pearson WS, Capewell S (2009) Trends in the prevalence of low risk factor burden for cardiovascular disease among United States adults. Circulation 120: 1181–1188. 19752328 10.1161/CIRCULATIONAHA.108.835728

[13] Office for National Statistics (2007). *Smoking and drinking among adults, 2007.*

[14] O’Flaherty M, Bishop J, Redpath A, McLaughlin T, Murphy D, et al (2009) Coronary heart disease mortality among young adults in Scotland in relation to social inequalities: time trend study. BMJ 339: b2613. 19602713 10.1136/bmj.b2613 PMC2714675

[15] Department for Communities and Local Government (2007) The English Indices of Deprivation 2007. (http://communities.gov.uk/documents/communities/pdf/733520.pdf).

[16] Norman P, Boyle P, Rees P (2005) Selective migration, health and deprivation: a longitudinal analysis. Soc Sci Med 60: 2755–2771. 15820585 10.1016/j.socscimed.2004.11.008

[17] Norman P, Simpson L, Sabater A (2008) ‘Estimating with confidence’ and hindsight: new UK small area population estimates for 1991. Population, Space and Place 14: 449–472.

[18] Mackenbach JP, Kunst AE (1997) Measuring the magnitude of socio-economic inequalities in health: an overview of available measures illustrated with two examples from Europe. Soc Sci Med 44: 757–771. 9080560 10.1016/s0277-9536(96)00073-1

[19] Allender S, Scarborough P, O’Flaherty M, Capewell S (2008) Patterns of coronary heart disease mortality over the 20th century in England and Wales: possible plateaus in the rate of decline. BMC Public Health 8: 148. 18452595 10.1186/1471-2458-8-148 PMC2386471

[20] Vaartjes I, O’Flaherty M, Grobbee DE, Bots ML, Capewell S (2011) Coronary heart disease mortality trends in the Netherlands 1972–2007. Heart 97: 569–573. 21282134 10.1136/hrt.2010.206565

[21] Bertuccio P, Levi F, Lucchini F, Chatenoud L, Bosetti C, et al (2011) Coronary heart disease and cerebrovascular disease mortality in young adults: recent trends in Europe. Eur J Cardiovasc Prev Rehabil 18: 627–634. 21521726 10.1177/1741826710389393

[22] McCartney D, Scarborough P, Webster P, Rayner M (2012) Trends in social inequalities for premature coronary heart disease mortality in Great Britain, 1994–2008: a time trend ecological study. BMJ Open 2: e000737. 10.1136/bmjopen-2011-000737 PMC3378944 22710128

[23] Mackenbach JP, Bos V, Andersen O, Cardano M, Costa G, et al (2003) Widening socioeconomic inequalities in mortality in six Western European countries. Int J Epidemiol 32: 830–837. 14559760 10.1093/ije/dyg209

[24] Jemal A, Ward E, Anderson RN, Murray T, Thun MJ (2008) Widening of socioeconomic inequalities in U.S. death rates, 1993–2001. PLoS One 3: e2181. 18478119 10.1371/journal.pone.0002181 PMC2367434

[25] Goldacre M (1993) Cause-specific mortality: understanding uncertain tips of the disease iceberg. J Epidemiol Community Health 47: 491–496. 8120506 10.1136/jech.47.6.491 PMC1059865

[26] Office for National Statistics (2002) Results of the ICD-10 bridge coding study, England and Wales, 1999. Health Stat Q 14: 75–83.

[27] Gregory IN (2009) Comparisons between geographies of mortality and deprivation from the 1900s and 2001: spatial analysis of census and mortality statistics. BMJ 339: b3454. 19744974 10.1136/bmj.b3454 PMC2741565

[28] Leyland AH, Lynch JW (2009) Why has mortality from coronary heart disease in young adults levelled off? BMJ 339: b2515. 19602712 10.1136/bmj.b2515

[29] Davey Smith G, Hart C, Watt G, Hole D, Hawthorne V (1998) Individual social class, area-based deprivation, cardiovascular disease factors, and mortality: the Renfrew and Paisley study. J Epidemiol Community Health 52: 399–405. 9764262 10.1136/jech.52.6.399 PMC1756721

[30] Ramsay SE, Morris RW, Lennon LT, Wannamethee SG, Whincup PH (2008) Are social inequalities in mortality in Britain narrowing? Time trends from 1978 to 2005 in a population-based study of older men. J Epidemiol Community Health 62: 75–80. 18079337 10.1136/jech.2006.053207 PMC2709224

[31] Adams J (2006) White M (2006) Removing the health domain from the Index of Multiple Deprivation 2004– effect on measured inequalities in census measures of health. J Public Health (Oxf) 28: 379–83. 17065177 10.1093/pubmed/fdl061

[32] Hawkins N, Scholes S, Bajekal M, Love H, O’Flaherty M, et al.. (2011) Cardiovascular disease and inequalities: Reducing socioeconomic inequality in coronary disease treatments: The NHS finally triumphs? J Epidemiol Community Health 65:Suppl 2 A20–A21 doi:10.1136/jech.2011.143586.46.

[33] Bajekal M, Scholes S, Love H, Hawkins N, O’Flaherty M, et al (2012) Analysing recent socioeconomic trends in coronary heart disease mortality in England, 2000–2007: a population modelling study. PLoS Med 9: e1001237. 22719232 10.1371/journal.pmed.1001237 PMC3373639

[34] Scholes S, Bajekal M, Love H, Hawkins N, Raine R, et al (2012) Persistent socioeconomic inequalities in cardiovascular risk factors in England over 1994–2008: A time-trend analysis of repeated cross-sectional data. BMC Public Health 12: 129. 22333887 10.1186/1471-2458-12-129 PMC3342910

[35] Office for National Statistics (2007). Trends in Life Expectancy by social class 1972–2005. Accessed 10^th^ Jan 2013. http://www.ons.gov.uk/ons/rel/health-ineq/health-inequalities/trends-in-life-expectancy-by-social-class-1972-2005/index.html.

[36] Capewell S, Graham H (2010) Will cardiovascular disease prevention widen health inequalities? PLoS Med 7: e1000320. 20811492 10.1371/journal.pmed.1000320 PMC2927551

