# Supplementary material for: Unequal Trends in Coronary Heart Disease Mortality by Socioeconomic Circumstances, England 1982–2006: An Analytical Study
Source: PLoS One. 2013 Mar 20;8(3):e59608. doi: 10.1371/journal.pone.0059608 (PMC3603902; doi:10.1371/journal.pone.0059608)
Supplement: Text S1 — Implications of using a fixed IMD quintile allocation for small areas in England from 1981 to 2007. (DOCX) [file pone.0059608.s003.docx]

### Text S1: Implications of using a fixed IMD quintile allocation for small areas in England from 1981 to 2007.

Supporting information for:

### Unequal Trends in Coronary Heart Disease Mortality by Socioeconomic Circumstances, England 1982-2006: Analytical Study

Authors

Madhavi Bajekal^1*^, Shaun Scholes^1^, Martin O’Flaherty^2^, Rosalind Raine^1^, Paul Norman^3^, Simon Capewell^2^

^1^ Department of Applied Health Research, University College London, London, UK

^2^Institute of Psychology, Health and Society, University of Liverpool, Liverpool, UK.

^3^ School of Geography, University of Leeds, Leeds, UK

Corresponding author: Madhavi Bajekal

*^*^Email:* [*m.bajekal@ucl.ac.uk*](mailto:m.bajekal@ucl.ac.uk)

### Background

In this study we have used the composite score of the 2007 Index of Multiple Deprivation (IMD) to categorise Lower Super Output Areas (LSOAs) into equal quintile groups of areas. The IMD is the government’s current preferred indicator of deprivation in England. Its main strength is that unlike deprivation indices based on census data, the majority of the 33 indicators which underlie the composite score can be updated between the inter-censal period using routinely collected data. The IMD scores also provide a more granular and precise measure of local deprivation as they are based on LSOAs which, unlike electoral wards, are statistical units designed to contain roughly equal sized populations and capture similar 'neighbourhoods'. Furthermore, because LSOA boundaries remain fixed over time, the distortions caused by frequent changes in the boundaries of alternative units of aggregation are eliminated (e.g. electoral ward or local administrative districts) [1]. Hence, the index was so designed to allow regularly updated IMD scores to be used to monitor the ‘real’ underlying trends in area inequalities.

The IMD series was first released in 2000 (at electoral ward level). Subsequent updates in 2004, 2007 and 2010 were all produced at the LSOA level. The indices from 2004 onwards are highly inter-correlated; this was expected as they use very similar, though not the same, datasets and methods.

For the purpose of our analysis of mortality trends from 1982 to 2006, we used the IMD score closest to the end-point of our series – 2007 – to define our quintile groups with Q1 being the least deprived and Q5 the most deprived areas. The IMD quintile group membership of an area and its boundary remained fixed over the entire period of analysis, i.e. 1982-2006. This was partly for practical reasons - there was no equivalent composite score of multiple deprivation for LSOAs available prior to 2004 - and partly because the *relative* ranking of small areas in England is thought to have remained remarkably stable over long periods whatever measure of relative deprivation is used [2].

### Does area deprivation ranking remain stable over time?

Linking a time-series of health events to area types fixed for a single point in time is a common way of analysing change over time. Since areas may change, this is recognised as less than ideal. We know that over time some areas undergo ‘gentrification’ while others move down the deprivation ladder. Selective (net) population migration between quintile groups is also likely to have an impact on the average ‘healthiness’ or otherwise of areas. However, aggregated over a large number of similar LSOAs (c. 6,500 areas, with a combined population of about 6.5 million adults aged 35 and over) we would expect that the net effect of moves of individual LSOAs on the margins between quintile groups would have a minor impact. Hence, we tested our assumption of relative stability in quintile allocation across time and the potential scale of the ‘noise’.

Dr Paul Norman at the University of Leeds has previously analysed change in deprivation levels between 1991 and 2001 Censuses using the Townsend index of deprivation calculated at common ward geography [3]. The aim of this analysis was to look at absolute change over the decade. We, on the other hand, were interested in the stability or otherwise of the *relative* ranking in quintile allocation of wards over time. We therefore requested Dr Norman to share with us the Townsend scores he had calculated using data from three censuses – 1981, 1991 and 2001 – calculated on common ward geography across all three time periods.

We normalised the deprivation score in each time period to the England average so that scores reflected the ward’s relative ranking at each time point. Unlike LSOAs, because wards were of unequal population sizes (larger in inner-city deprived areas), we calculated population-weighted quintile groups such that each quintile had equal fifths of the population, not equal numbers of areas. On average, wards are about four times larger than LSOAs. In 2001 there were 7958 CAS (Census Area Statistics) wards in England with an average population of 6,174 persons (min=557 max=35,102); in contrast there were 32,482 LSOAs with an average population of 1,513 persons (min=1,000 max=6,537). Note there is a positive correlation of 0.88 (p<0.001) between Townsend scores calculated for LSOAs using 2001 Census data and the IMD for 2007.

Our analysis of change in the relative position of wards, as allocated to deprivation quintiles, was carried out using the Townsend deprivation scores for 1981, 1991 and 2001.

### Results

Table 1 below shows the transition matrix of the percentages of the 1981 population as distributed into deprivation quintiles based on 1981 score ranking and 2001 score ranking. Table 2 shows the equivalent quintile matrix for the transition between 1991 and 2001 rankings for 1991 population.

Table 1: Transition matrix of % population distribution categorised into quintiles by 1981 and 2001 Townsend deprivation scores, England

| 1981 deprivation quintiles |  | 2001 deprivation quintiles | | | | |  |
| --- | --- | --- | --- | --- | --- | --- | --- |
|  |  | Q1 | Q2 | Q3 | Q4 | Q5 | TOTAL |
|  | Q1 | 76 | 27 | 5 | 0 | 0 | 20 |
|  | Q2 | 21 | 53 | 27 | 3 | 0 | 20 |
|  | Q3 | 3 | 18 | 51 | 24 | 3 | 20 |
|  | Q4 | 1 | 1 | 17 | 56 | 21 | 20 |
|  | Q5 | 0 | 0 | 1 | 17 | 76 | 20 |
|  | TOTAL | 100 | 100 | 100 | 100 | 100 | 100 |
| Summary | |  |  |  |  |  |  |
| No change in quintile | | 76 | 53 | 51 | 56 | 76 | 62 |
| Moved up 1+quintile | | 24 | 20 | 18 | 17 | 0 | 15 |
| Moved down 1+quintile | | 0 | 27 | 31 | 27 | 24 | 22 |

In terms of population distribution, Table 1 shows that just over three-quarters (76%) of the population in 1981 who were living in either in the least or the most deprived fifths of wards remained in their respective top / bottom quintiles in 2001 - i.e. their position relative to national deprivation level remained unchanged. As we might expect, there was more movement in the intermediate quintile groups (up and down). Hence, the population in wards that retained their deprivation group categorisation over these two decades (i.e. 'on the diagonal') was 62%. The remainder were fairly evenly split: 15% moved up one or more quintile groups ('gentrification'), and 22% moved down the deprivation ladder.

Table 2: Transition matrix of % population distribution categorised into quintiles by 1991 and 2001 Townsend deprivation scores, England

| 1981 deprivation quintiles (1991?) |  | 2001 deprivation quintiles | | | | |  |
| --- | --- | --- | --- | --- | --- | --- | --- |
|  |  | Q1 | Q2 | Q3 | Q4 | Q5 | TOTAL |
|  | Q1 | 80 | 22 | 1 | 0 | 0 | 20 |
|  | Q2 | 18 | 63 | 20 | 0 | 0 | 20 |
|  | Q3 | 2 | 15 | 65 | 17 | 0 | 20 |
|  | Q4 | 0 | 1 | 13 | 71 | 13 | 20 |
|  | Q5 | 0 | 0 | 0 | 12 | 87 | 20 |
|  | TOTAL | 100 | 100 | 100 | 100 | 100 | 100 |
| Summary | |  |  |  |  |  |  |
| No change in quintile | | 80 | 63 | 65 | 71 | 87 | 73 |
| Moved up 1+quintile | | 20 | 16 | 14 | 12 | 0 | 12 |
| Moved down 1+quintile | | 0 | 22 | 21 | 17 | 13 | 15 |

The equivalent table for transitions between 1991 and 2001 is markedly more stable than that between 1981 and 2001 (Table 2). Changes in the 1980s included a large reduction in non-home ownership as people took advantage of the ‘right-to-buy’ their council rented property (39.6% non-home ownership in 1981, 29.7% in 1991 and 28% in 2001). 80% of the 1991 population living in the most advantaged quintile and 87% of those in the most deprived retained their respective position. Overall, about three-quarters (73%) of England’s population did not change quintile position between 1991 and 2001. Of the remainder, 12% of the population moved up one or more quintile groups, and 15% moved down the quintile grouping.

These results demonstrate that the top and bottom fifths of the deprivation distribution remained fairly stable over the two decades of the study, particularly so at the extreme ends of the distribution whereby in general non-deprived wards remain so as do deprived wards. However, as we might expect, the consistency of the match deteriorated over time.

It is therefore plausible to assume that the quintile match between LSOAs over the 25-year period would have been at least as stable as for CAS wards, and possibly more so.

### Selective migration and it impact on inequality trends.

Numerous studies have shown that there is a consistent relationship between health and deprivation: poorer areas have the worst health outcomes, with health improving as deprivation falls. This relationship is partly because areas are socially segregated (i.e. the area composition effect) and partly because of differences in the physical environment, resources and facilities between areas (or the contextual effects). However it should be borne in mind that not all socially disadvantaged people live in deprived areas, and vice-versa. Since area-based deprivation measures capture both the contextual and compositional aspects of deprivation, they may be a more reliable measure of socioeconomic circumstances than disadvantage measured between groups based on individual social position alone.

Recently, a number of studies examining the health-deprivation relationship have explored the possibility that at least part of the explanation for the persistence of inequality relates to selective migration of healthy people to less deprived areas and for either sicker people to move to poorer areas or the relative immobility of sicker people relative to healthy out-migrants.

Norman and colleagues (2005)l used the closed population sample of the UK Longitudinal Study (LS) to examine the health effects of net internal migration between relatively deprived and affluent areas between 1971, 1981 and 1991 [4]. To control for initial poor-health selection, those who reported being sick or disabled in 1971 were excluded from the analysis. In general they found that those who were downwardly mobile had poorer health than their origin group, but better health than their destination group. Conversely, those who went up the social ladder had health intermediate between their group of origin and the more advantaged group they joined. Those who remained in the top quintile across all censuses had the best health outcomes; and those who stayed in the bottom quintile had the worst health.

These findings would suggest that selective migration would tend to reduce the (cross-sectional) health gap between rich and poor areas. However the researchers found the opposite: selective mobility increased the health gap [4-6].

The authors explored this apparent paradox further and showed that this was because of the distribution in the relative numbers of those who moved into an area, those who moved out and those who stayed put in the same quintile group. For example, in the most deprived quintile, mortality rates increase because those moving out of the quintile (largest group) had better health than those who moved into it (next biggest group); who in turn had better health than the 'stayers' (the smallest group). In contrast, in the least deprived quintile, the out-migrants were the largest group and had poorer health than both the new in-migrants and the stayers. The net effect of these moves therefore resulted in the widening of the mortality inequalities between the most and least deprived quintiles.

Is the impact of selective migration material to cross-sectional analysis of trends in inequalities in mortality? Norman and colleagues concluded that unlike health status measures such as limiting long-standing illness, the impact of mobility on mortality was **not** significant and that the estimated deprivation gradients will not be 'exaggerated to a significant degree' [4]. Furthermore, the dominant flow is by relatively healthy people aged 20-59 moving from more to less deprived areas, rather than the older age groups who are the focus of our research.

Over time, geographical patterns of inequality are maintained and often exaggerated. Where areas change their level of deprivation or where people’s deprivation circumstances change, then there are likely to be concomitant changes in health. The majority of change (as with social mobility) is in the ‘middle ground’ rather than in the extremes. Wholesale change is rare.

**In summary: the majority of small areas in England have remained in their quintile group over the 25-year period of our analysis. Furthermore, although selective migration of healthy people to better-off areas is a factor, net migration would have some, but not a significant, impact on the analysis of trends in inequalities we report.**

### References:

1. Norman P, Rees P & Boyle P (2003) Achieving data compatibility over space and time: creating consistent geographical zones. *International Journal of Population Geography.* 9(5): 365-386.

2. Gregory I (2009) Comparisons between geographies of mortality and deprivation from the 1900s and 2001: spatial analysis of census and mortality statistics. BMJ 339:b3454.

3. Norman P (2010) Identifying change over time in small area socio-economic deprivation. *Appl Spatial Analysis and Policy* 3: 107-138.

4. Norman P, Boyle P, Rees P (2005) Selective migration, health and deprivation: a longitudinal analysis. *Social Science and Medicine* 60: 2755-71.

5. Boyle P, Norman P (2009) Chapter 19: ‘*Migration and Health’*. In T Brown, S McLafferty, C Moon eds. **A companion to health and medical geography**. Wiley-Blackwell, Oxford, UK.

6. Boyle P, Norman P & Popham F (2009) Social mobility: evidence that it can widen health inequalities. *Social Science & Medicine* 68(10): 1835-1842,
